# Novel piperidine derivatives as colchicine binding site inhibitors induce apoptosis and inhibit epithelial-mesenchymal transition against prostate cancer PC3 cells

**DOI:** 10.1080/14756366.2020.1783664

**Published:** 2020-06-26

**Authors:** Dong-Jun Fu, Si-Meng Liu, Jia-Jia Yang, Jun Li

**Affiliations:** a Modern Research Center for Traditional Chinese Medicine, School of Chinese Materia Medica, Beijing University of Chinese Medicine, Beijing, People’s Republic of China; b Department of Gastroenterology, the Fifth Affiliated Hospital of Zhengzhou University, Zhengzhou, People’s Republic of China; c Department of Pharmacy, People’s Hospital of Zhengzhou, Zhengzhou, People’s Republic of China

**Keywords:** Colchicine binding site inhibitors, piperidine, apoptosis, epithelial-mesenchymal transition

## Abstract

Tubulin polymerisation inhibitors that target colchicine binding site were powerful anticancer agents. Although along the years many colchicine binding site inhibitors (CBSIs) have been reported, few piperidine derivatives were identified as CBSIs. In this regard, we focussed efforts on the piperidine as a promising chemotype to develop potent CBSIs. Herein, novel piperidine derivatives were synthesised and evaluated for their antiproliferative activities. Among them, compound **17a** displayed powerful anticancer activity with the IC_50_ value of 0.81 µM against PC3 cells, which was significantly better than 5-fluorouracil. It could inhibit tubulin polymerisation binding at the colchicine site and inhibit the tumour growth *in vitro* and *in vivo*. Further biological studies depicted that **17a** suppressed the colony formation, induced apoptosis, and inhibited epithelial-mesenchymal transition against PC3 cells. These results revealed that compound **17a** is a promising colchicine binding site inhibitor for the treatment of cancer and it is worthy of further exploitation.

## Graphical Abstract

**Figure:**
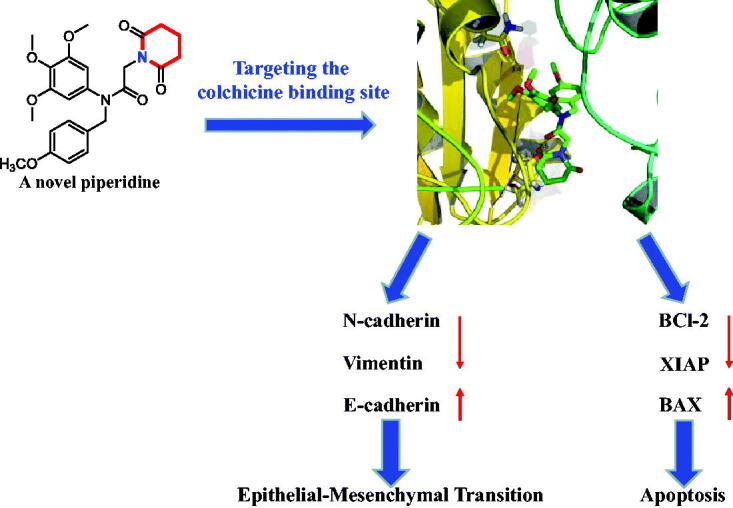

## 1. Introduction

Microtubules are dynamic cytoskeletal polymers of tubulin, which involved in essential cellular functions, such as cell growth, mitosis, motility, intracellular transport, and division^1^^,^^2^. Because of the important role of microtubules, they have become an important target for the design of new anticancer agents^3^^,^^4^. Microtubule-targeting agents interact with tubulin through at least four binding sites: the laulimalide site, paclitaxel site, vinca site, and colchicine site^5^. In contrast to the paclitaxel site and vinca site, the colchicine binding site is located between the *α*- and *β*-monomers of the *α,β*-tubulin heterodimer^6^. Colchicine binding site inhibitors (CBSIs) exhibit the potently inhibitory effects of tumour cell proliferation and metastasis^7^. Therefore, an increased attention has been focussed on the discovery of CBSIs.

**Figure 1.**
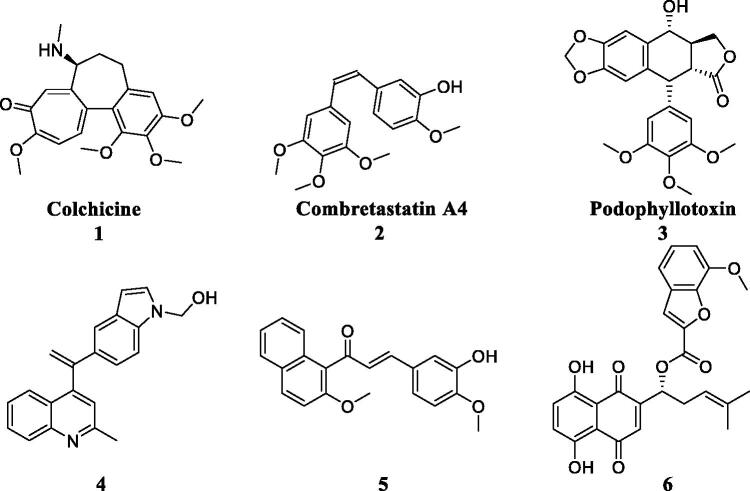
Reported colchicine binding site inhibitors (CBSIs).

In recent years, some colchicine binding site inhibitors have been reported as anticancer agents (Figure 1)^8^. Colchicine (**1**), combretastatin A4 (**2**) and podophyllotoxin (**3**) as anticancer agents could induce apoptosis and cell death by targeting colchicine binding site^9^. Quinoline-indole derivative **4** disrupted cell microtubule networks and arrested the cell cycle at G2/M phase against K562 cells^10^. Chalcone **5** as a colchicine binding site inhibitor displayed the potent antiproliferative activity with an IC_50_ value of 1.42 µM against MCF7 cells^11^. Shikonin-benzo[*b*]furan **6** could regulate the expression of apoptosis related proteins in HT29 cells by targeting the colchicine binding site^12^. Although these CBSIs demonstrated potent antitumor activity against various cancer cell lines, there clinical trials were halted due to serious side effects, low solubility and low bioavailability^13^. As to overcome the limitations, it is imperative to discover novel colchicine binding site inhibitors.

**Figure 2.**
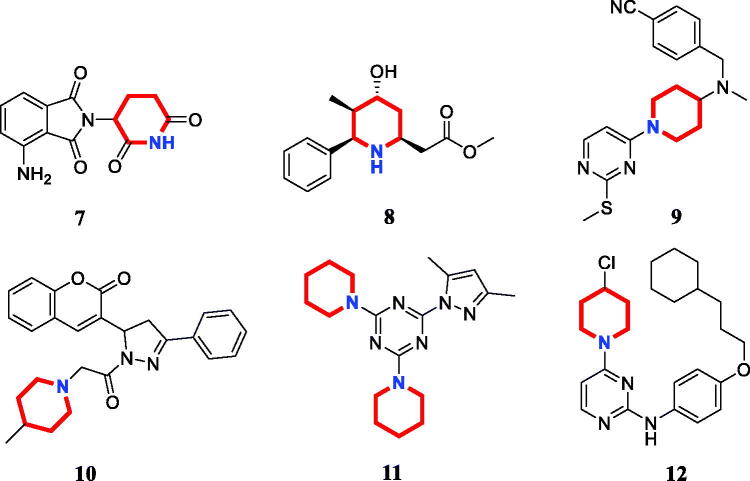
Chemical structures of anticancer piperidine derivatives.

Piperidine is a privileged scaffold in the field of drug discovery which provides numerous opportunities in exploring this moiety as an anticancer agent by acting on various receptors of utmost importance (Figure 2)^14^. Pomalidomide (**7**) was a FDA approved drug to treat multiple myeloma by inhibiting TNF_α_^15^. Piperidine **8** displayed the antiproliferative activity with IC_50_ values from 5.4 µM to 8.5 µM against A549, MCF7, DU145, and HeLa cell lines^16^. Piperidine derivative **9** was a novel human heat shock protein 70 inhibitor for the treatment of drug-resistant tumours^17^. 5-Phenyl-*N*-piperidine ethanone containing 4,5-dihydropyrazole derivative **10** occupied high antiproliferative activities against SGC-7901, MGC-803 and Bcap-37 cells^18^. Piperidine **11** affected the cell viability and induced G2/M phase cell cycle arrest in K562 cells^19^. Piperidine **12** was a selective inhibitor against triple-negative breast cancer cell line MDA-MB-468^20^.

Our research group has long been involved in designing new colchicine binding site inhibitors with the aim to discover various anticancer agents^21–24^. In this work, we report the synthesis and anticancer evaluation of novel piperidine derivatives as colchicine binding site inhibitors. Importantly, their effects on apoptosis and epithelial-mesenchymal transition (EMT) were also revealed.

## 2. Experimental

### 2.1. General procedure for the synthesis of compound 16

To a solution of 3,4,5-trimethoxyaniline (5 mmol, 1.0 eq) and 1-(chloromethyl)-4-methoxybenzene (5.5 mmol, 1.1 eq) in *N*,*N*-dimethylformamide (20 ml) was added triethylamine (7.5 mmol, 1.5 eq) at room temperature. The mixture was stirred at room temperature for eight hours. Upon completion, ethyl acetate and water were added. The aqueous layer was extracted with ethyl acetate for several times and the combined organic layers were evaporated to give the crude product **15**. 2-Chloroacetyl chloride (3 mmol, 1.5 eq) and potassium carbonate (3 mmol, 1.5 eq) were added to the solution of crude product **15** (2 mmol, 1eq) in acetone. After stirring at room temperature for four hours, the reaction mixture was concentrated to remove then treated with a solution of ethyl acetate and H_2_O. The organic layer was washed with brine, dried over anhydrous sodium sulphate, and then concentrated to provide compound **16** without purification. The NMR analyses were described in Supplemental data.

### 2.2. General procedure for the synthesis of piperidine and pyrrolidine derivatives 17a∼17g

Cyclamine (2 mmol, 2 eq) was added to the mixture of compound **16** (1 mmol, 1 eq) and sodium hydroxide (1.5 mmol, 1.5 eq) in acetone (5 ml). The mixture was stirred at room temperature for 12 h. Upon completion, the solvent was removed under reduced pressure, the residue was extracted with dichloromethane, washed with brine and concentrated under reduced pressure. The residue was purified with column chromatography (hexane: EtOAc = 9:1) to obtain analogues **17a∼17g**.

#### 2–(2,6-Dioxopiperidin-1-yl)-N-(4-methoxybenzyl)-N-(3,4,5-trimethoxyphenyl) acetamide (17a)

Yield: 62%. White solid, m.p.:1 5 6 ∼ 158 °C. ^1^H NMR (400 MHz, CDCl_3_) δ 7.05 (d, *J* = 8.5 Hz, 2H), 6.74 (d, *J* = 8.6 Hz, 2H), 6.22 (s, 2H), 4.69 (s, 2H), 4.26 (s, 2H), 3.76 (s, 3H), 3.71 (s, 3H), 3.66 (s, 6H), 2.64 (t, *J* = 6.5 Hz, 4H), 1.99–1.90 (m, 2H). ^13 ^C NMR (100 MHz, CDCl_3_) δ 171.35, 165.46, 158.02, 152.59, 136.88, 135.29, 129.35, 128.31, 112.72, 104.74, 59.92, 55.19, 54.26, 51.65, 40.23, 31.45, 16.02. HRMS (*m/z*) [M + H]^+^ calcd for C_24_H_29_N_2_O_7_, 457.1975; found, 457.1979.

#### 2–(7,9-Dioxo-8-azaspiro[4.5]decan-8-yl)-N-(4-methoxybenzyl)-N- (3,4,5-trimethoxyphenyl)acetamide (17 b)


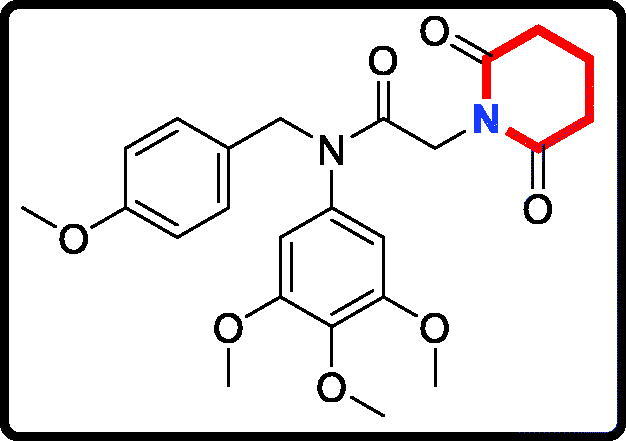


Yield: 52%. White solid, m.p.:9 5 ∼ 97 °C. ^1^H NMR (400 MHz, CDCl_3_) δ 7.04 (d, *J* = 8.5 Hz, 2H), 6.73 (d, *J* = 8.5 Hz, 2H), 6.21 (s, 2H), 4.70 (s, 2H), 4.25 (s, 2H), 3.76 (s, 3H), 3.71 (s, 3H), 3.65 (s, 6H), 2.58 (s, 4H), 1.66 (t, *J* = 6.8 Hz, 4H), 1.54 (d, *J* = 6.4 Hz, 4H). ^13 ^C NMR (100 MHz, CDCl_3_) δ 171.11, 165.45, 158.01, 152.57, 136.86, 135.27, 129.37, 128.32, 112.71, 104.77, 59.91, 55.18, 54.25, 51.52, 43.35, 40.22, 38.59, 36.65, 23.10. HRMS (*m/z*) [M + H]^+^ calcd for C_28_H_35_N_2_O_7_, 511.2444; found, 511.2448.

#### 2–(4,4-Dimethyl-2,6-dioxopiperidin-1-yl)-N-(4-methoxybenzyl)-N- (3,4,5-trimethoxyphenyl)acetamide (17c)


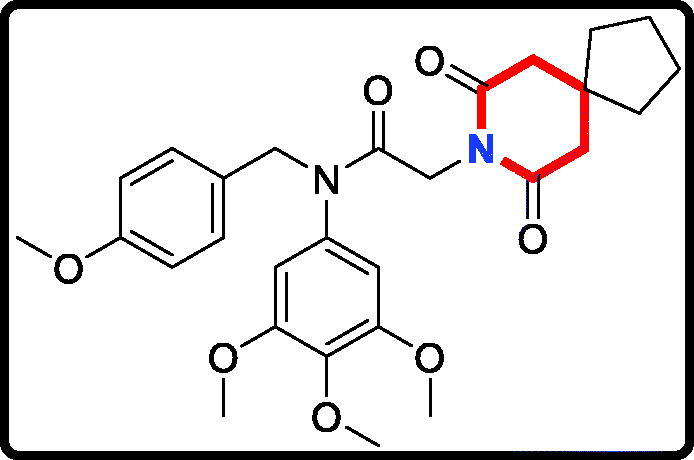


Yield: 46%. White solid, m.p.:1 4 1 ∼ 143 °C. ^1^H NMR (400 MHz, CDCl_3_) δ 7.04 (d, *J* = 8.5 Hz, 2H), 6.73 (d, *J* = 8.6 Hz, 2H), 6.21 (s, 2H), 4.70 (s, 2H), 4.26 (s, 2H), 3.76 (s, 3H), 3.71 (s, 3H), 3.65 (s, 6H), 2.49 (s, 4H), 1.11 (s, 6H). ^13 ^C NMR (100 MHz, CDCl_3_) δ 170.88, 165.52, 158.01, 152.58, 136.87, 135.26, 129.37, 128.31, 112.71, 104.76, 59.91, 55.18, 54.25, 51.53, 45.03, 40.13, 28.32, 26.77. HRMS (*m/z*) [M + H]^+^ calcd for C_26_H_33_N_2_O_7_, 485.2288; found, 485.2294.

#### N-(4-methoxybenzyl)-2-(pyrrolidin-1-yl)-N-(3,4,5-trimethoxyphenyl)acetamide (17d)


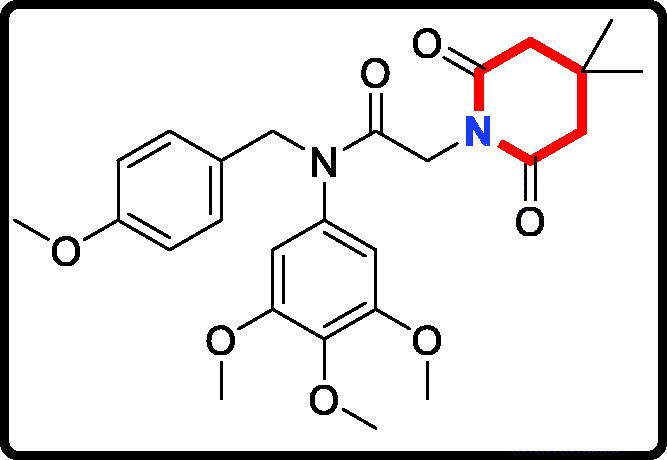


Yield: 75%. White solid, m.p.:9 4 ∼ 96 °C. ^1^H NMR (400 MHz, CDCl_3_) δ 7.08 (d, *J* = 8.6 Hz, 2H), 6.72 (d, *J* = 8.5 Hz, 2H), 6.05 (s, 2H), 4.70 (s, 2H), 3.78 (s, 3H), 3.71 (s, 3H), 3.63 (s, 6H), 3.02 (s, 2H), 2.51 (s, 4H), 1.70 (s, 4H). ^13 ^C NMR (100 MHz, CDCl_3_) δ 168.54, 157.97, 152.39, 136.63, 136.08, 129.59, 128.92, 112.60, 104.82, 59.94, 56.07, 55.12, 54.25, 53.23, 51.24, 22.66. HRMS (*m/z*) [M + H]^+^ calcd for C_23_H_31_N_2_O_5_, 415.2233; found, 415.2237.

#### N-(4-methoxybenzyl)-2-(piperidin-1-yl)-N-(3,4,5-trimethoxyphenyl)acetamide (17e)


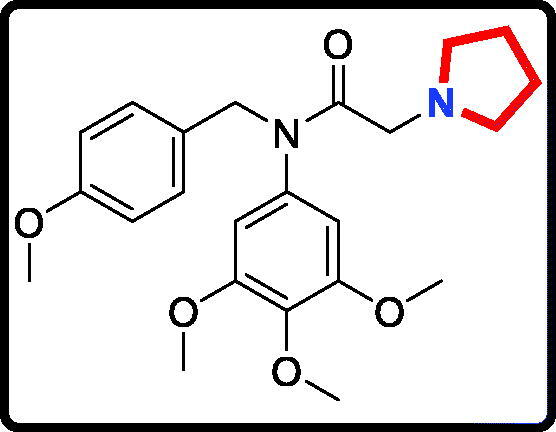


Yield: 61%. White solid, m.p.:7 5 ∼ 77 °C. ^1^H NMR (400 MHz, CDCl_3_) δ 7.08 (d, *J* = 8.5 Hz, 2H), 6.72 (d, *J* = 8.5 Hz, 2H), 6.04 (s, 2H), 4.69 (s, 2H), 3.78 (s, 3H), 3.71 (s, 3H), 3.63 (s, 6H), 2.85 (s, 2H), 2.35 (s, 4H), 1.57–1.42 (m, 4H), 1.32 (s, 2H). ^13 ^C NMR (100 MHz, CDCl_3_) δ 168.38, 157.96, 152.34, 136.60, 136.20, 129.57, 128.93, 112.59, 104.88, 59.95, 59.30, 55.13, 54.25, 53.61, 51.28, 24.86, 22.98. HRMS (*m/z*) [M + H]^+^ calcd for C_24_H_33_N_2_O_5_, 429.2389; found, 429.2392.

#### N-(4-methoxybenzyl)-2–(3-methylpiperidin-1-yl)-N-(3,4,5-trimethoxyphenyl)acetamide (17f)


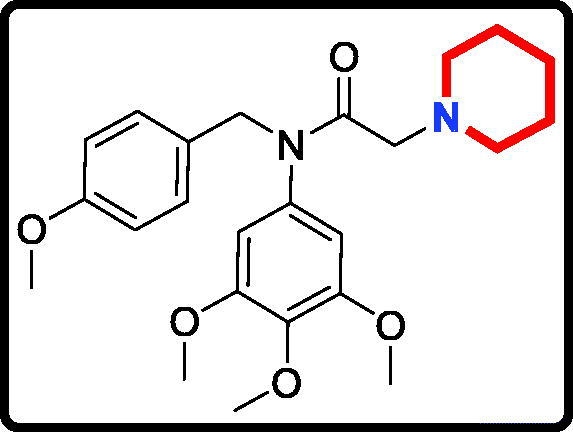


Yield: 80%. White solid, m.p.:7 2 ∼ 74 °C. ^1^H NMR (400 MHz, CDCl_3_) δ 7.08 (d, *J* = 8.5 Hz, 2H), 6.72 (d, *J* = 8.4 Hz, 2H), 6.04 (s, 2H), 4.69 (s, 2H), 3.78 (s, 3H), 3.71 (s, 3H), 3.63 (s, 6H), 2.86 (s, 2H), 2.74 (s, 2H), 1.85 (s, 1H), 1.59 (d, *J* = 10.3 Hz, 3H), 1.51 (s, 2H), 0.75 (s, 1H), 0.75 (d, *J* = 5.6 Hz, 3H). ^13 ^C NMR (100 MHz, CDCl_3_) δ 168.44, 157.97, 152.34, 136.60, 136.20, 129.58, 128.93, 112.59, 104.89, 61.00, 59.95, 59.09, 55.13, 54.25, 53.04, 51.27, 31.65, 30.06, 24.50, 18.64. HRMS (*m/z*) [M + H]^+^ calcd for C_25_H_35_N_2_O_5_, 443.2546; found, 443.2548.

#### 2–(3,5-Dimethylpiperidin-1-yl)-N-(4-methoxybenzyl)-N-(3,4,5-trimethoxyphenyl)acetamide (17 g)


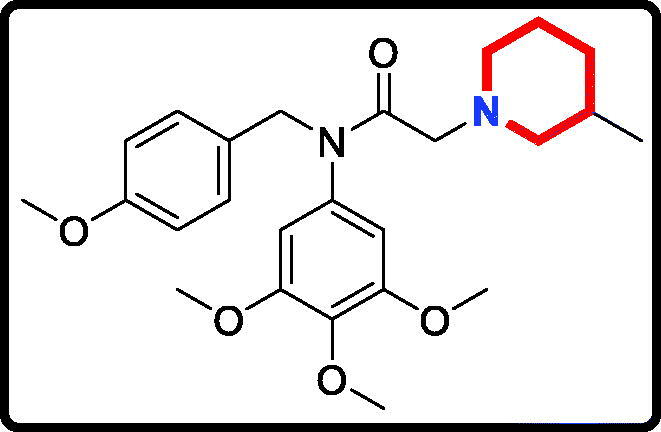


Yield: 62%. White solid, m.p.:9 6 ∼ 98 °C. ^1^H NMR (400 MHz, CDCl_3_) δ 7.08 (d, *J* = 8.4 Hz, 2H), 6.72 (d, *J* = 8.4 Hz, 2H), 6.04 (s, 2H), 4.70 (s, 2H), 3.78 (s, 3H), 3.71 (s, 3H), 3.63 (s, 6H), 2.87 (s, 2H), 2.74 (d, *J* = 9.5 Hz, 2H), 1.61 (s, 2H), 1.58 (s, 1H), 1.48 (s, 2H), 0.74 (d, *J* = 6.3 Hz, 6H), 0.40 (q, *J* = 11.8 Hz, 1H). ^13 ^C NMR (100 MHz, CDCl_3_) δ 168.43, 157.98, 152.35, 136.61, 136.15, 129.60, 128.92, 112.59, 104.88, 60.52, 59.95, 58.79, 55.12, 54.25, 51.27, 40.79, 30.02, 18.51. HRMS (*m/z*) [M + H]^+^ calcd for C_26_H_37_N_2_O_5_, 457.2702; found, 457.2706.

### 2.3. Mtt assay

MGC803 (gastric cancer cells), PC3 (prostate cancer cells) and MCF7 (breast cancer cells) were obtained from the Chinese Academy of Sciences (Shanghai, China). Dimethyl sulfoxide was used to mix the piperidine derivatives **17a∼17g** to different concentrations (0.1 µM, 0.5 µM, 1 µM, 2 µM, 4 µM, 8 µM, 16 µM, 32 µM, and 64 µM). Tumour cells were seeded into 96-well plates in 100 µL of culture medium. Then, cells were treated in triplicate with a gradient concentration of **17a∼17g** and incubated at 37 °C, 5% CO_2_ for 48 h. For all cell lines, MTT (thiazolyl blue tetrazolium bromide) assay was performed to measure the antiproliferative activity^25^.

### 2.4. Colony formation assay

PC3 cells were seeded in a 6-well plate and incubated for 24 h, then treated with **17a** at different concentrations (0, 0.125 µM, 0.25 µM and 0.5 µM). After seven days, the culture medium was removed and cells were washed with phosphate buffer saline. Then, the system was fixed with 4% paraformaldehyde and stained with 0.1% crystal violet. The cells image was captured with the camera (Jinghua Co. Ltd, Beijing, China).

### 2.5. Dapi staining

PC3 cells were seeded in a 6-well plate and incubated for 24 h, then treated with **17a** at different concentrations (0, 0.5 µM and 1 µM). After 48 h incubation, remove the medium, wash the cells with phosphate buffer saline gently, and fix the cells with 4% paraformaldehyde for 20 min. Cells were then stained with 4′,6-diamidino-2-phenylindole (DAPI) in the dark for 20 min.

### 2.6. Cell apoptosis assay

PC3 cells were seeded in a 6-well plate and incubated for 24 h, then treated with **17a** at different concentrations (0, 0.5 µM and 1 µM) for 48 h. Then, cells were harvested and the AnnexinV-FITC/PI kit (Jiangsu KeyGEN BioTECH Corp., Ltd, Zhilan Road, Jiangning District, Nanjing, China) was used according to the manufacturer’s instructions to perform the apoptosis assay.

### 2.7. Wound healing

PC3 cells were seeded in a 6-well plate and incubated for 24 h. The cell surface was scratched using a pipet tip. Then, the cells were cultured with fresh medium containing 1% foetal bovine serum and different concentrations of **17a** (0, 0.5 µM and 1 µM) for 24 h.

### 2.8. Migration assay

PC3 cells were seeded in a Transwell plate (CORNING, Corning City, State of New York, USA). 1% heat-inactivated foetal bovine serum and 20% foetal bovine serum were added into upper and lower chamber separately. Different concentrations of **17a** (0, 0.5 µM and 1 µM) were added in the chambers. After the incubation for 48 h, remove the medium and wash the chambers with phosphate buffer saline. The migrating cells were fixed with methanol for 20 min and stained with Hoechst-33258 (Innochem, Beijing, China). Each chamber was photographed using Thermo Fisher Cellomics High Content System (Heqi Co. Ltd, Shanghai, China).

### 2.9. *In vitro* tubulin polymerisation assay

5.6 mg/ml tubulin was resuspended in PEM buffer (containing 80 mmol/L PIPES, 1 mmol/L EGTA, 0.5 mmol/L MgCl_2_, 1 mmol/L ATP and 10.2% (v/v) glycerol). The system was incubated with **17a** (0, 1 µM, 2 µM and 4 µM) and colchicine on ice. The reaction was monitored by a spectrophotometer (JB-750, Jiangsu, China) in absorbance at 420 nm at 37 °C every minute (excitation wavelength is 340 nm).

### 2.10. Ebi competition assay

*N*,*N*’-ethylenebis(iodoacetamide) (EBI) was purchased from Innochem company (Beijing, China). PC3 cells were seeded in a 6-well plate and incubated for 24 h. Cells were first incubated with compound **17a**, colchicine (15 µM) or dimethyl sulfoxide for 2 h and afterward treated with EBI (100 µM) for 2 h. Then, the cells were harvested and lysed to perform western blotting analysis.

### 2.11. Western blot analysis

PC3 cells were seeded in a 6-well plate and incubated for 24 h, then treated with **17a** at different concentrations (0, 0.5 µM, 1 µM and 1.5 µM). After the the incubation for 48 h, PC3 cells were harvested and lysed. Protein lysates were resolved by SDS-PAGE and transferred to nitrocellulose membrane. The membrane was incubated with appropriate antibodies at 4 °C for 12 h in 5% skimmed milk. After conjugated with secondary antibodies, the detection of proteins was carried out with an ECL Western Blotting Substrate Kit (Amyjet Scientific, Wuhan, China).

### 2.12. Docking studies

Piperidine derivative **17a** was selected to do molecular docking study. For the receptor preparation, the PDB code (1SA0) was downloaded from the Protein Data Bank. The docking studies were performed by Autodock 4.2 (The Scripps Research Institute, San Diego, California, USA).

### 2.13. *In vivo* anti-tumour activity

Animals were treated according to the protocols established by the ethics committee of Zhengzhou University and the *in vivo* experiments were carried out in accordance with the approved guidelines and approved by the ethics committee of Zhengzhou University. BALB/c nude mice were purchased from Hunan Slack Scene of Laboratory Animal Co. Ltd. (Hunan, China). Prostate cancer PC3 xenograft model was established in BALB/c mice. The tumour-bearing mice were randomised into three groups (six mice in each group) and subcutaneous injection with normal saline or compound **17a** (50 mg/kg) or colchicine (0.25 mg/kg) for 21 days. Compound **17a** was firstly resolved with the mixture of ethyl alcohol and castor oil, and then diluted in normal saline. Tumour size was determined by calliper measurement. The tumour volume was calculated using the ellipsoid volume formula (Length × Width^2^/2).

### 2.14. Statistical evaluation

Data were presented as means ± SD. Statistical analyses were performed by SPSS 17.0 and GraphPad.

## 3. Results and discussion

### 3.1. Chemistry

**Scheme 1.**
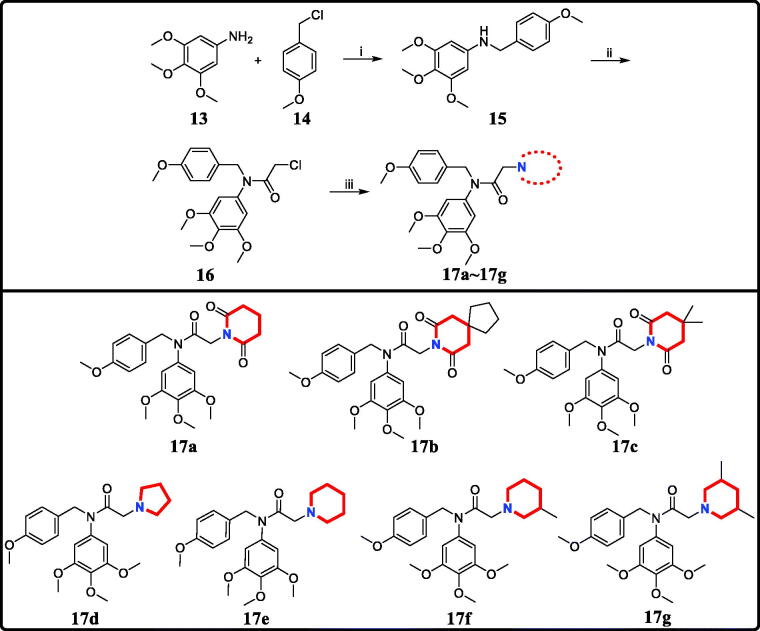
Reagents and conditions: (i) *N*,*N*-dimethylformamide, triethylamine; (ii) 2-chloroacetyl chloride, K_2_CO_3_, acetone; (iii) Piperidines or pyrrolidine, NaOH, acetone.

The target derivatives **17a**∼**17g** were synthesised from the starting material 3,4,5-trimethoxyaniline in a sequence of reactions and characterised by NMR and HRMS. Nucleophilic substitution reaction of 3,4,5-trimethoxyaniline with 1-(chloromethyl)-4-methoxybenzene afforded the secondary amine **15**, which was reacted with 2-chloroacetyl chloride leading to the key intermediate **16** (Scheme 1). Compound **16** reacted with piperidines and pyrrolidine in the presence of sodium hydroxide to give derivatives **17a**∼**17g**.

### 3.2. Antiproliferative activity

To discover novel antitumor agents, we evaluated the antiproliferative activity of all piperidine and pyrrolidine derivatives **17a∼17g** against three cancer cell lines PC3 (prostate cancer cell line), MGC803 (gastric cancer cell line) and MCF7 (breast cancer cell line) using the 3–(4,5-dimethyl-2-thiazolyl)-2,5-diphenyl-2-*H*-tetrazolium bromide (MTT) assay. Based on the previous reference 5,26-fluorouracil and colchicine as well-known antitumor agents were selected as the reference drugs in this work.

The antiproliferative activity results of candidate derivatives **17a∼17g** against all three cancer cell lines were shown in Table 1. Novel piperidine derivatives **17a∼17c** exhibited more potent antiproliferative activity against all selected cancer cell lines with IC_50_ values from 0.81 µM to 9.37 µM than 5-fluorouracil. Among all target derivatives, compound **17a** displayed the best antiproliferative activity with the IC_50_ value of 1.09 µM, 0.81 µM, and 1.30 µM against MGC803 cells, PC3 cells and MCF7 cells, respectively. These antiproliferative activity results indicate that piperidine-2,6-dione fragment might display potent anticancer activity *in vitro* and could be used as the leading unit to design more similar derivatives.

**Table 1.** Antiproliferative activity of candidate derivatives **17a∼17g**.

^a^ Antiproliferative activity was assayed by the exposure for 48 h.

In addition, candidate derivatives attaching different cyclamine units (pyrrolidine, piperidine, 3-methylpiperidine, and 3,5-dimethylpiperidine) were synthesised and evaluated for their antiproliferative activity. By this way, the molecular diversity of piperidine derivatives were explored. Changing the piperidine unit (**17e**) to a pyrrolidine unit (**17d**) led to a decrease of the inhibitory activity against all the tested cell lines, indicating that the piperidine fragment was unfavourable for the antiproliferative activity. When the 3-methylpiperidine group (**17f**) and the 3,5-dimethylpiperidine group (**17 g**) were replaced by a piperidine group (**17e**), the antiproliferative activity was improved against all cancer cell lines. All these modifications revealed that the piperidine scaffold was important for their inhibitory activity.

### 3.3. Compound 17a inhibited the colony formation and cell viability

**Figure 3.**
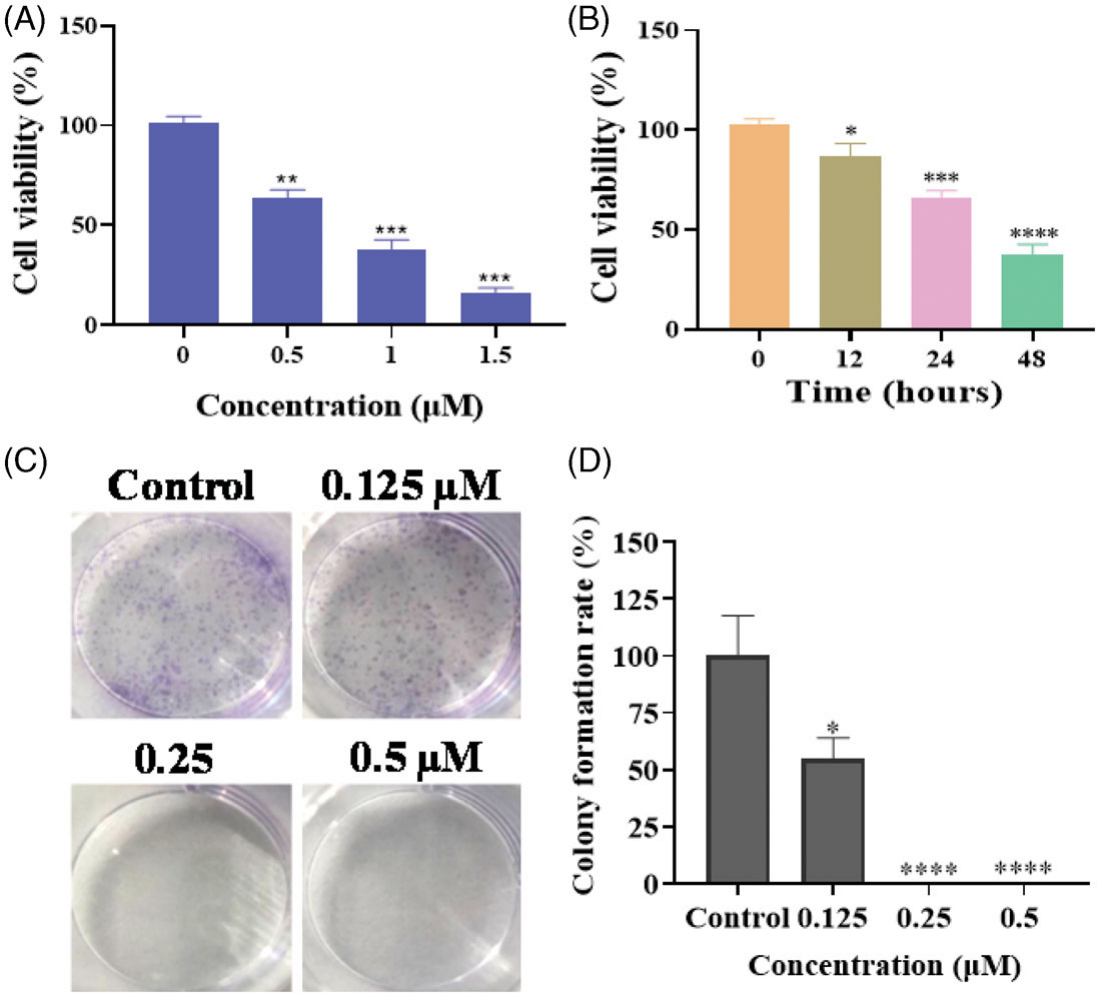
(A) The cell viability of PC3 cells with the treatment of compound **17a** at different concentrations for 48 h. (B) The cell viability of PC3 cells with the treatment of compound **17a** at 1 μM for different hours. (C) Colony formation of PC3 cells with the treatment of **17a** at various concentrations for 7 days. (D) Colony formation rate. *p < .05 *verse* control, **p < .01 *verse* control, ***p < .001 *verse* control and ****p < .0001 *verse* control.

Based on the screening activity results of all synthetic derivatives, compound **17a** as the most potent compound was chosen to perform colony formation to investigate whether piperidine derivatives could inhibit the proliferation of tumour cells. As shown in Figure 3(A,B), the cell viability of PC3 cells were decreased with the treatment of compound **17a** in a time-dependent manner and a concentration-dependent manner.

PC3 cells with the treatment of **17a** exhibited fewer and smaller colonies compared to the control (Figure 3(C,D)), which indicated that piperidine derivative **17a** could significantly inhibit the proliferation of PC3 cells in a concentration-dependent manner.

### 3.4. Compound 17a induced morphological changes and apoptosis

**Figure 4.**
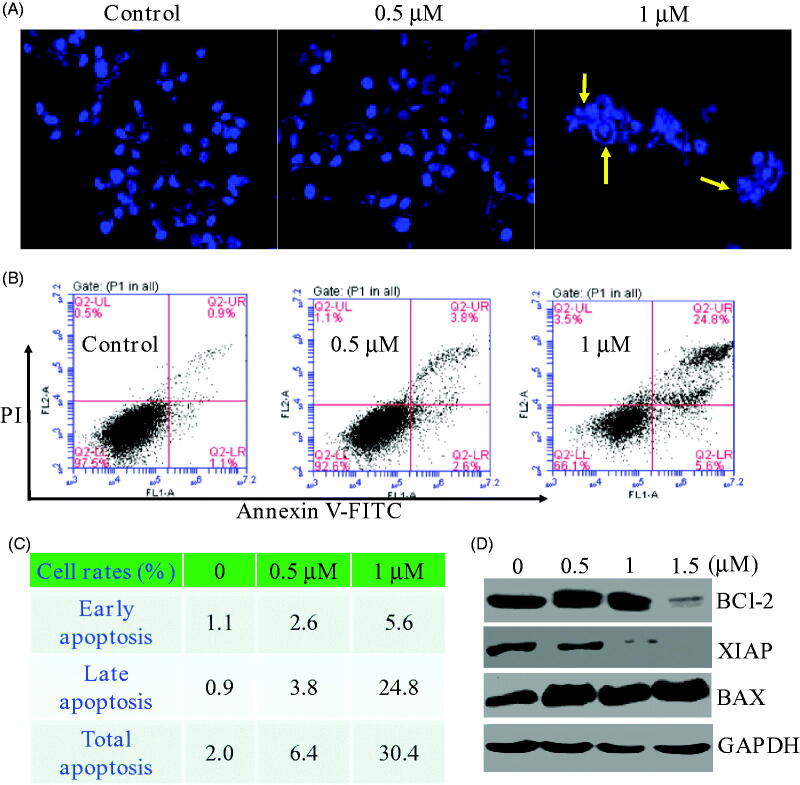
(A) Morphological changes analysis with DAPI staining after 48 h of compound **17a** in PC3 cells. (B&C) Quantitative analysis of apoptotic cells using Annexin V-FITC/PI double staining. (D) Expression levels of **17a** on apoptosis-related proteins.

Inspired by the potent inhibition of the piperidine derivative **17a** against PC3 cells, we then investigated whether **17a** was able to induce morphological changes. After being incubated with the piperidine derivative **17a** for 48 h at different concentrations (0 µM, 0.5 µM and 1 µM), the morphological changes of PC3 cells were recorded. As shown in Figure 4(A), significant changes of cell morphology such as rounding up and cell debris were observed at the high concentration (yellow arrows). The effects of compound **17a** on the apoptosis was also investigated using the propidium iodide (PI) and Annexin V-FITC. As illustrated in Figure 4(B,C), compound **17a** induced apoptosis of PC3 cells in a concentration-dependent manner. Specifically, the percentage of apoptotic cells was about 2.0% for the control group. When treated with the high concentration (1 µM) of compound **17a**, around 30.4% of apoptosis rate was observed. To further investigate the mechanism of apoptosis, western blot analysis was performed to explore the expression levels of apoptosis-related proteins in PC3 cells. As shown in Figure 4(D), the treatment of PC3 cells with **17a** resulted in decreased expression levels of BCl-2 and XIAP in a concentration-dependent manner.

Meanwhile, the expression of BAX increased accordingly. Therefore, compound **17a** could induce morphological changes and apoptosis in PC3 cells.

### 3.5. Compound 17a inhibited the epithelial-mesenchymal transition in PC3 cells

**Figure 5.**
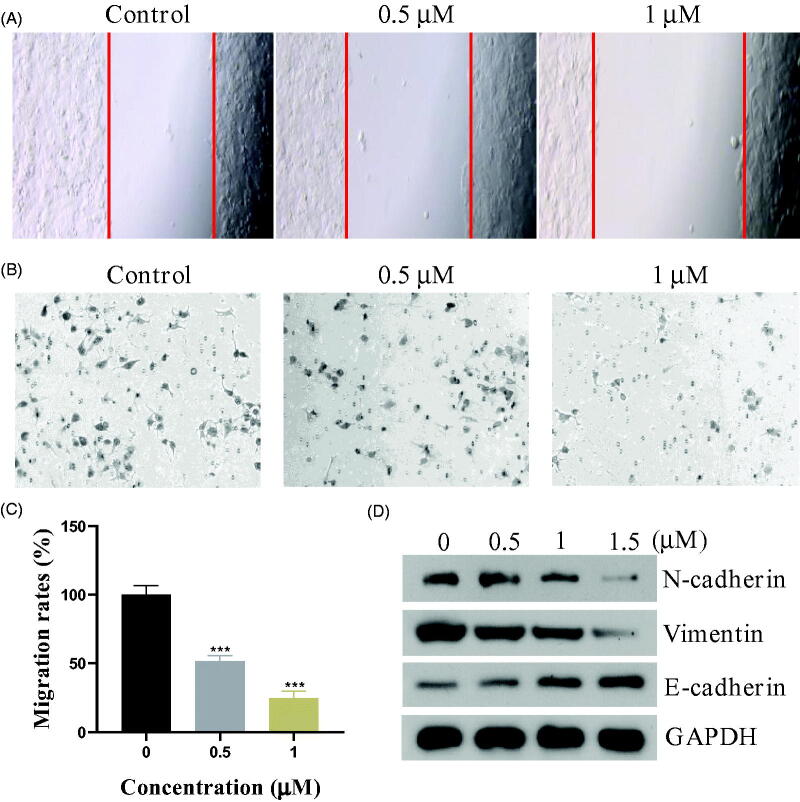
(A) Wound healing assay. (B&C) The migration ability of PC3 cells after the treatment of compound **17a**. (D) Expression levels of N-cadherin, Vimentin, and E-Cadherin.

The epithelial-mesenchymal transition (EMT) is a key developmental programme that is often activated during cancer migration and metastasis^27^. In this work, we evaluated the migration ability of PC3 cells by wound healing and migration assay. As shown in Figure 5(A), the piperidine derivative **17a** inhibited the wound healing obviously. The migration assay (Figure 5(B,C)) also demonstrated that compound **17a** hindered the PC3 cells migration through the biological membrane. We also examined the expression of the typical proteins of EMT process by western blot experiments. Figure 5(D) showed that compound **17a** could upregulate the epithelial cells’ biomarker E-Cadherin while the mesenchymal cells’ biomarkers, N-Cadherin and Vimentin, were down-regulated correspondingly. All these results indicated that compound **17a** inhibited the epithelial-mesenchymal transition in PC3 cells.

### 3.6. Compound 17a was a novel colchicine binding site inhibitor

**Figure 6.**
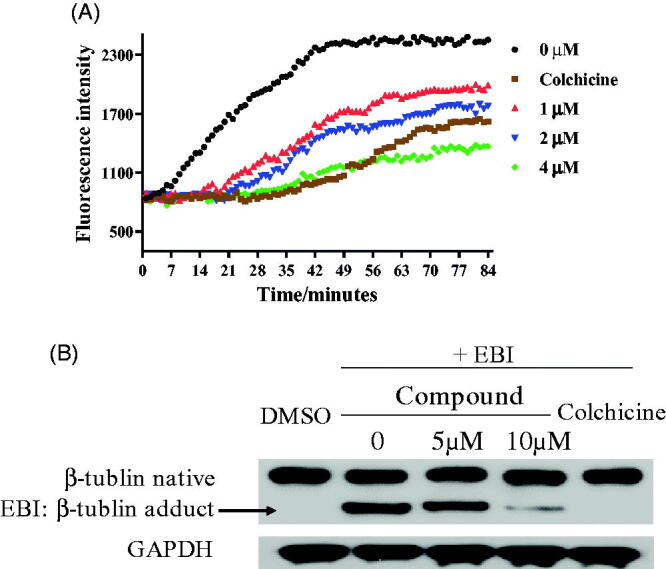
(A) Tubulin polymerisation inhibitory activity of **17a**. (B) EBI competition assay on PC3 cells.

The tubulin polymerisation inhibition activity *in vitro* of compound **17a** was evaluated. When tubulin was incubated with the piperidine derivative **17a** at different concentrations (1 µM, 2 µM and 4 µM), the increased tendency of the fluorescence intensity was obviously slowed down. The IC_50_ value of compound **17a** was 2.03 µM against tubulin (Figure 6(A)). It revealed that the piperidine derivative **17a** was a novel tubulin polymerisation inhibitor. In the *N*,*N*’-ethylenebis(iodoacetamide) (EBI) assay, compound **17a** at 10 µM prevented the formation of EBI: β-tubulin adduct comparing with DMSO and EBI treatment. These results in Figure 6(B) indicated that the piperidine derivative **17a** directly bind to the colchicine binding site.

### 3.7. Molecular docking studies of compound 17a

**Figure 7.**
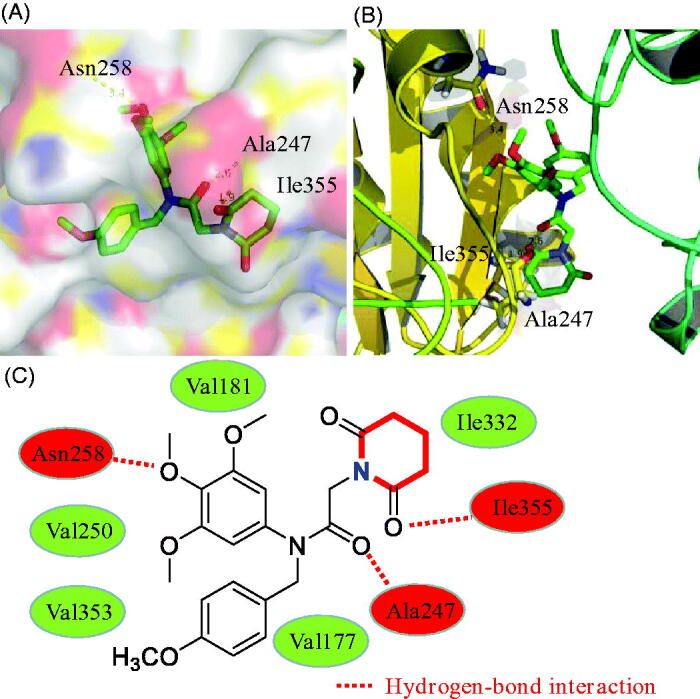
(A,B) 3D binding models of compound **17a** in the active sites of tubulin. (C) 2D binding models and hydrogen-bond interactions.

In this work, molecular docking methodologies were also used to explore any molecular interactions between the piperidine derivative **17a** and tubulin. We used the autodock software as an automated tool to perform docking and selected PDB code 1SA0. As shown in Figure 7, 3,4,5-trimethoxyphenyl ring, amide group, and piperidine-2,6-dione formed three hydrogen bonds with residues Ala247, Asn258 and Ile355, respectively. In addition, derivative **17a** formed hydrophobic interactions with residues Val250, Val353, Val181, Val177 and Ile332. These docking results of compound **17a** may provide a basis for further optimisation.

### 3.8. *In vivo* antitumor study of compound 17a

**Figure 8.**
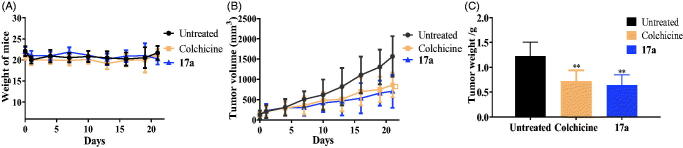
*In vivo* antitumor effects after the treatment of normal saline (untreated group), colchicine (0.25 mg/kg), compound **17a** (50 mg/kg). (A) Weight of mice. (B) Tumour volume. (C) Tumour weight of mice. **p < .01 *verse* control.

Due to the best inhibitory activity of compound **17a** against PC3 cells, we also evaluated the *in vivo* antitumor effects of compound **17a** on xenograft models bearing PC3 cells by the sub-cutaneous implantation. After the treatment of compound **17a**, the weight of mice, the tumour weight and the tumour volume were measured and recorded. As shown in Figure 8, the piperidine derivative **17a** inhibited tumour growth, while the body weight was almost unchanged, indicating the antitumor efficacy and low toxicity.

## 4. Conclusions

In the present study, we investigated the anticancer effects *in vitro* and *in vivo* of the piperidine derivative **17a**. Our findings showed that compound **17a** could induce apoptosis and inhibit the epithelial-mesenchymal transition progress in PC3 cells as a novel colchicine binding site inhibitor. Thus, the piperidine derivative **17a** could be a lead compound for further anti-prostate cancer drug discovery.

## Supplementary Material

Supplemental Material
