# Supplementary material for: Novel piperidine derivatives as colchicine binding site inhibitors induce apoptosis and inhibit epithelial-mesenchymal transition against prostate cancer PC3 cells
Source: J Enzyme Inhib Med Chem. 2020 Jun 26;35(1):1403–13. doi: 10.1080/14756366.2020.1783664 (PMC7646549; doi:10.1080/14756366.2020.1783664)
Supplement: Supplemental Material [file IENZ_A_1783664_SM8025.pdf]

Novel piperidine derivatives as colchicine binding site inhibitors induce apoptosis and inhibit epithelial-mesenchymal transition against prostate cancer PC3 cells

Dong-Jun Fu[a], Si-Meng Liu[b], Jia-Jia Yang[c], Jun Li[a*]

[a]Modern Research Center for Traditional Chinese Medicine, School of Chinese Materia Medica, Beijing University of Chinese Medicine, Beijing 100029, People's Republic of China

[b]Department of Gastroenterology, the Fifth Affiliated Hospital of Zhengzhou University, Zhengzhou 450052, People's Republic of China

[c]Department of Pharmacy, People's Hospital of Zhengzhou, Zhengzhou 450000, People's Republic of China

[*]Corresponding author: drlj666@163.com (Jun Li)

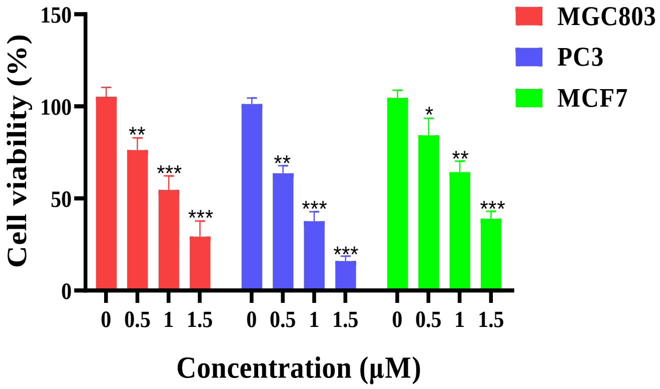

Figure 1s. The effects of **17a** in reducing cell viabilities of all selected cell lines (MGC803, PC3 and MCF7) for 48 hours.

2-(2,6-Dioxopiperidin-1-yl)-N-(4-methoxybenzyl)-N-(3,4,5-trimethoxyphenyl)acetamide (17a)

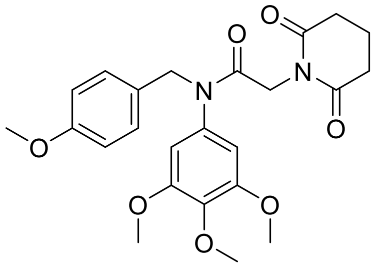

Yield: 62%. White solid, m.p.:156~158 °C. ^{1}H NMR (400 MHz, $CDCl_3$) δ 7.05 (d, *J* = 8.5 Hz, 2H), 6.74 (d, *J* = 8.6 Hz, 2H), 6.22 (s, 2H), 4.69 (s, 2H), 4.26 (s, 2H), 3.76 (s, 3H), 3.71 (s, 3H), 3.66 (s, 6H), 2.64 (t, *J* = 6.5 Hz, 4H), 1.99 – 1.90 (m, 2H). ^{13}C NMR (100 MHz, $CDCl_3$) δ 171.35, 165.46, 158.02, 152.59, 136.88, 135.29, 129.35, 128.31, 112.72, 104.74, 59.92, 55.19, 54.26, 51.65, 40.23, 31.45, 16.02. HRMS (m/z) $[M + H]^+$ calcd for $C_{24}H_{29}N_2O_7$, 457.1975; found, 457.1979.

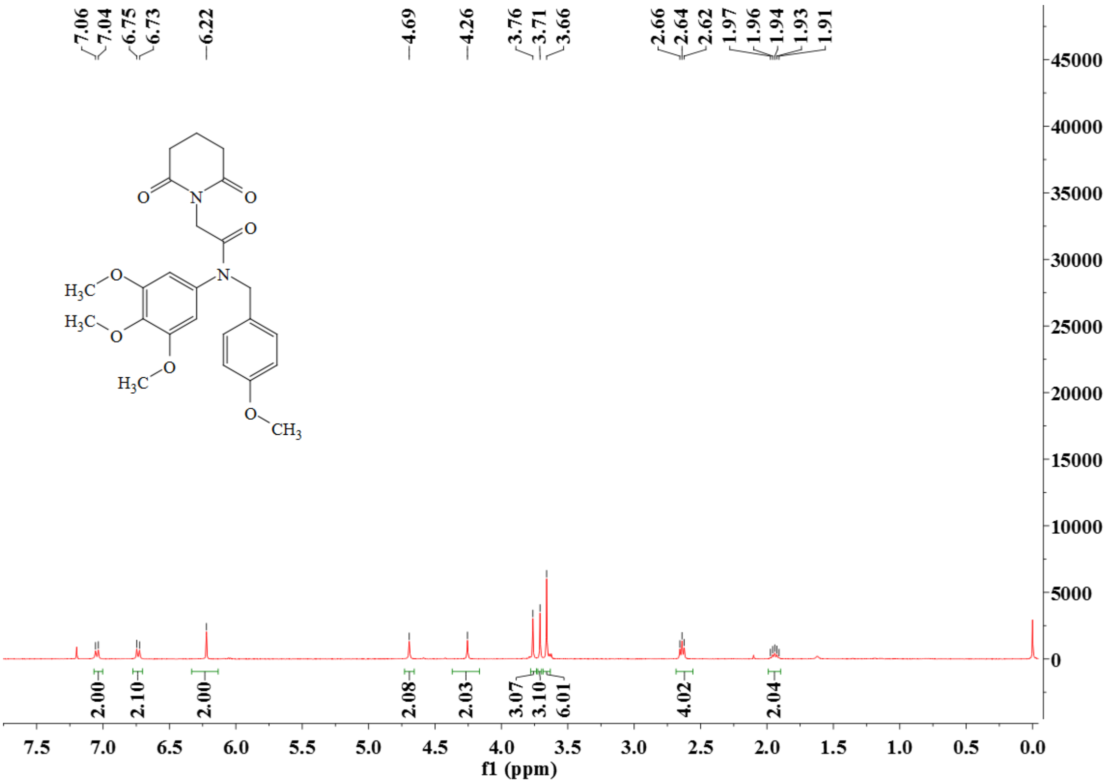

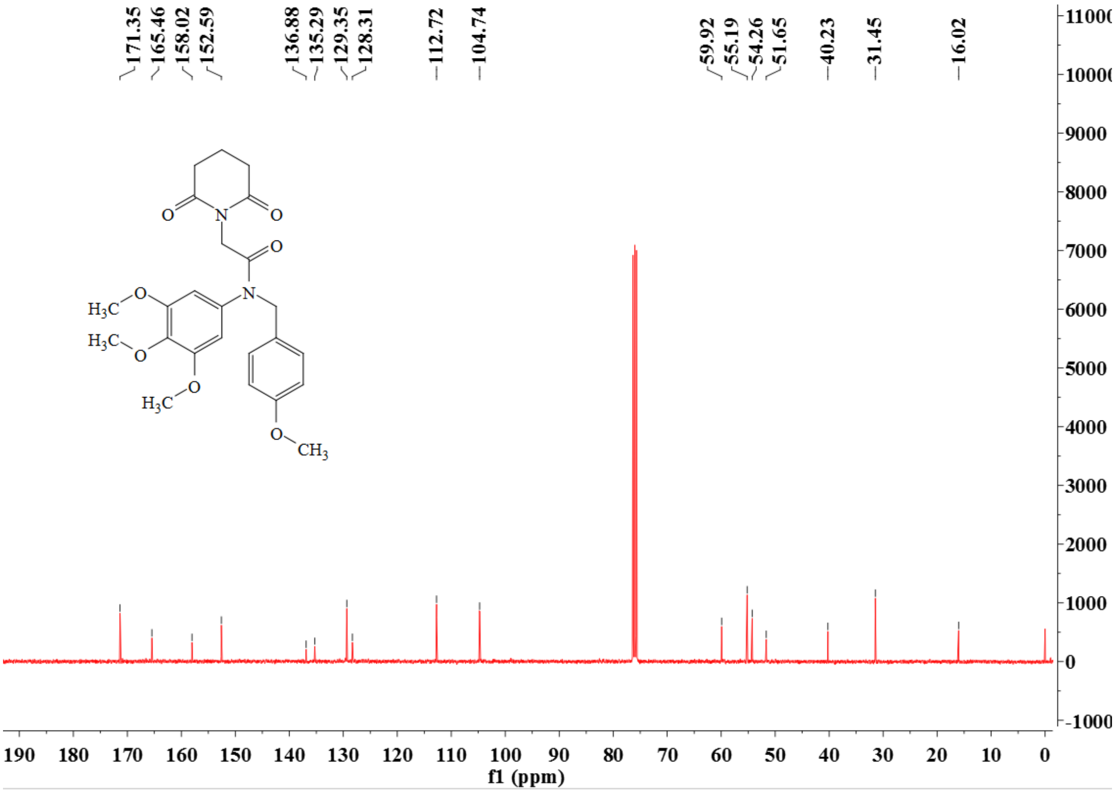

2-(7,9-Dioxo-8-azaspiro[4.5]decan-8-yl)-N-(4-methoxybenzyl)-N-(3,4,5-trimethoxyphenyl)acetamide (17b)

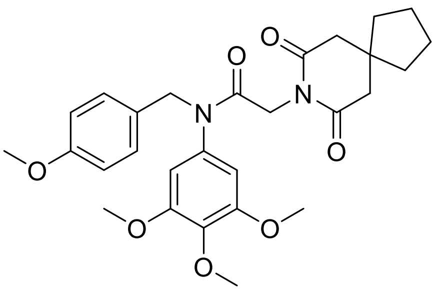

Yield: 52%. White solid, m.p.:95~97 ºC. ^{1}H NMR (400 MHz, $CDCl_3$) δ 7.04 (d, *J* = 8.5 Hz, 2H), 6.73 (d, *J* = 8.5 Hz, 2H), 6.21 (s, 2H), 4.70 (s, 2H), 4.25 (s, 2H), 3.76 (s, 3H), 3.71 (s, 3H), 3.65 (s, 6H), 2.58 (s, 4H), 1.66 (t, *J* = 6.8 Hz, 4H), 1.54 (d, *J* = 6.4 Hz, 4H). ^{13}C NMR (100 MHz, $CDCl_3$) δ 171.11, 165.45, 158.01, 152.57, 136.86, 135.27, 129.37, 128.32, 112.71, 104.77, 59.91, 55.18, 54.25, 51.52, 43.35, 40.22, 38.59, 36.65, 23.10. HRMS (m/z) $[M + H]^+$ calcd for $C_{28}H_{35}N_2O_7$, 511.2444; found, 511.2448.

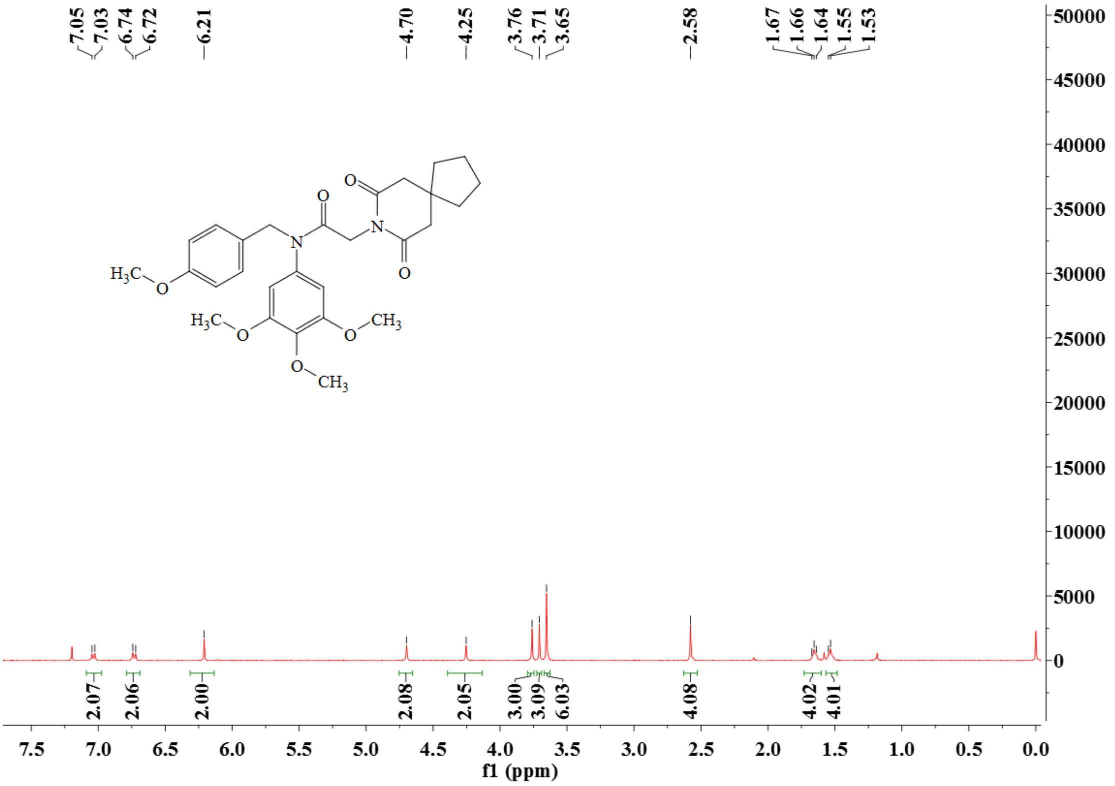

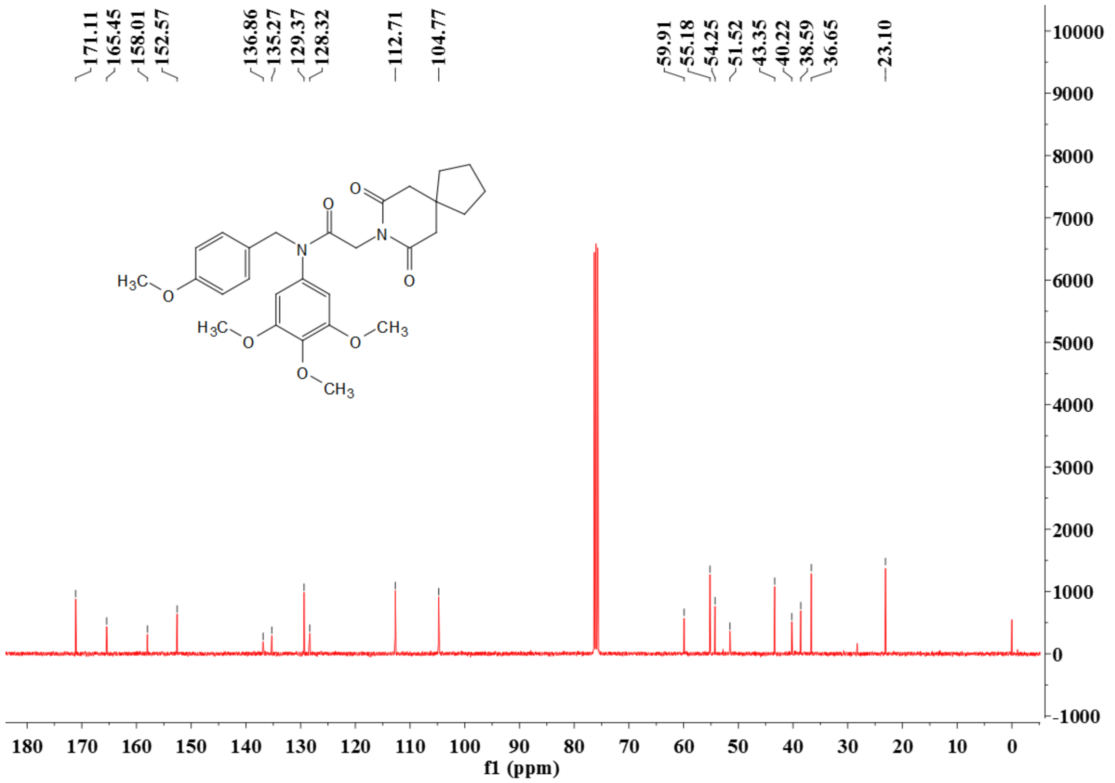

2-(4,4-Dimethyl-2,6-dioxopiperidin-1-yl)-N-(4-methoxybenzyl)-N-(3,4,5-trimethoxyphenyl)acetamide (17c)

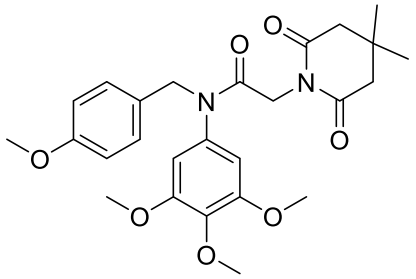

Yield: 46%. White solid, m.p.:141~143 ^{o}C. ^{1}H NMR (400 MHz, CDCl$_3$) δ 7.04 (d, *J* = 8.5 Hz, 2H), 6.73 (d, *J* = 8.6 Hz, 2H), 6.21 (s, 2H), 4.70 (s, 2H), 4.26 (s, 2H), 3.76 (s, 3H), 3.71 (s, 3H), 3.65 (s, 6H), 2.49 (s, 4H), 1.11 (s, 6H). ^{13}C NMR (100 MHz, CDCl$_3$) δ 170.88, 165.52, 158.01, 152.58, 136.87, 135.26, 129.37, 128.31, 112.71, 104.76, 59.91, 55.18, 54.25, 51.53, 45.03, 40.13, 28.32, 26.77. HRMS (m/z) $[M + H]^+$ calcd for $C_{26}H_{33}N_2O_7$, 485.2288; found, 485.2289.

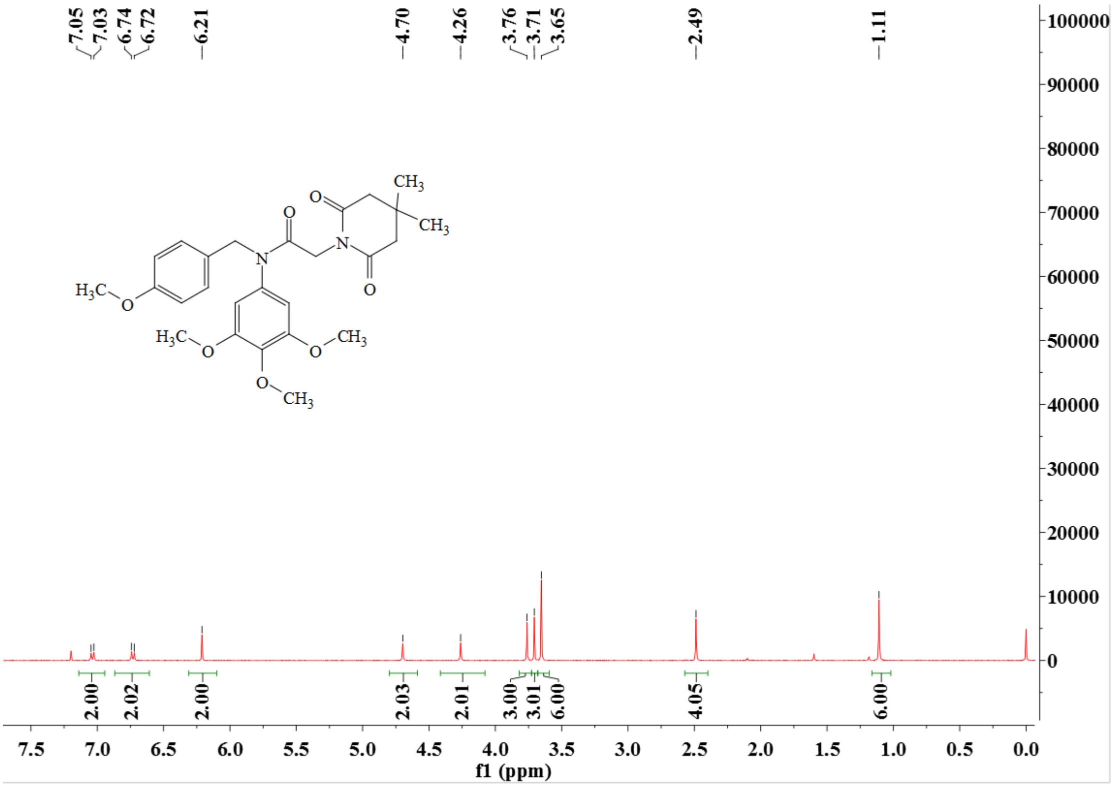

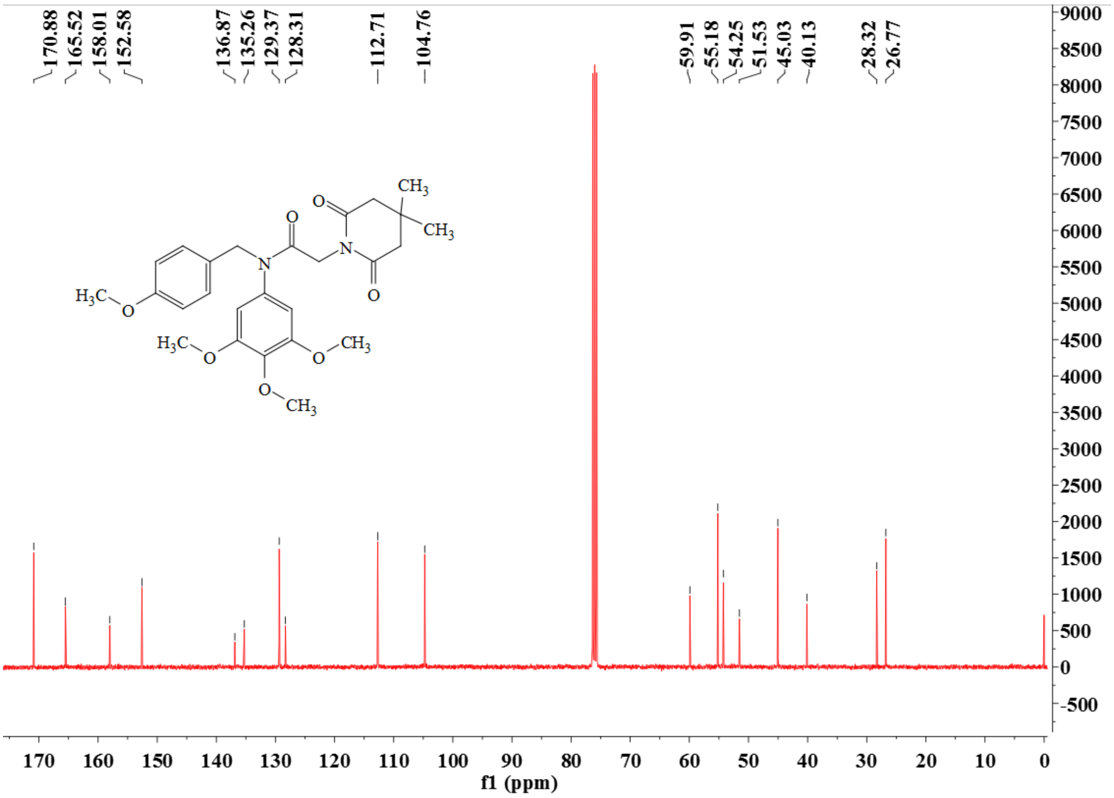

N-(4-methoxybenzyl)-2-(pyrrolidin-1-yl)-N-(3,4,5-trimethoxyphenyl)acetamide (17d)

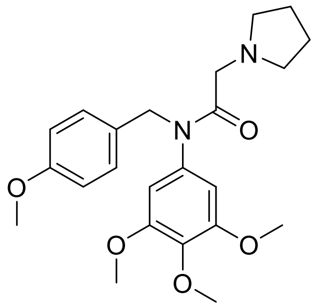

Yield: 75%. White solid, m.p.:94~96 ºC. ^{1}H NMR (400 MHz, $CDCl_3$) δ 7.08 (d, *J* = 8.6 Hz, 2H), 6.72 (d, *J* = 8.5 Hz, 2H), 6.05 (s, 2H), 4.70 (s, 2H), 3.78 (s, 3H), 3.71 (s, 3H), 3.63 (s, 6H), 3.02 (s, 2H), 2.51 (s, 4H), 1.70 (s, 4H). ^{13}C NMR (100 MHz, $CDCl_3$) δ 168.54, 157.97, 152.39, 136.63, 136.08, 129.59, 128.92, 112.60, 104.82, 59.94, 56.07, 55.12, 54.25, 53.23, 51.24, 22.66. HRMS (m/z) $[M + H]^+$ calcd for $C_{23}H_{31}N_2O_5$, 415.2233; found, 415.2237.

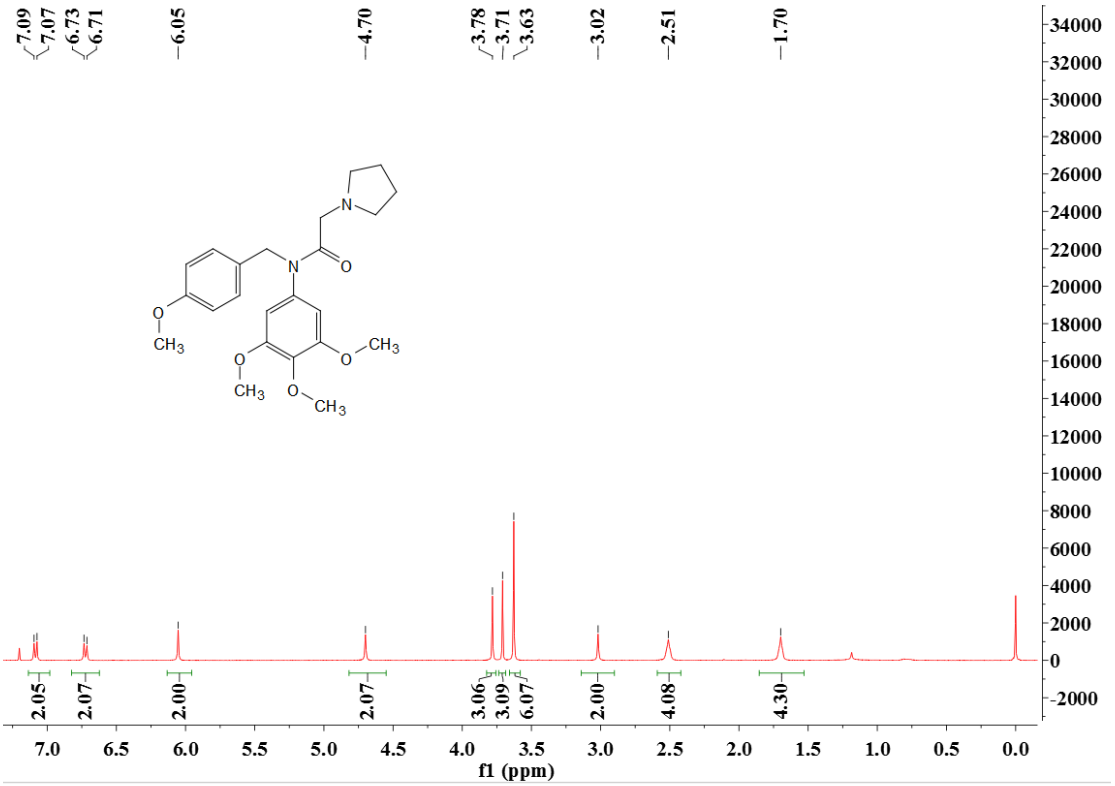

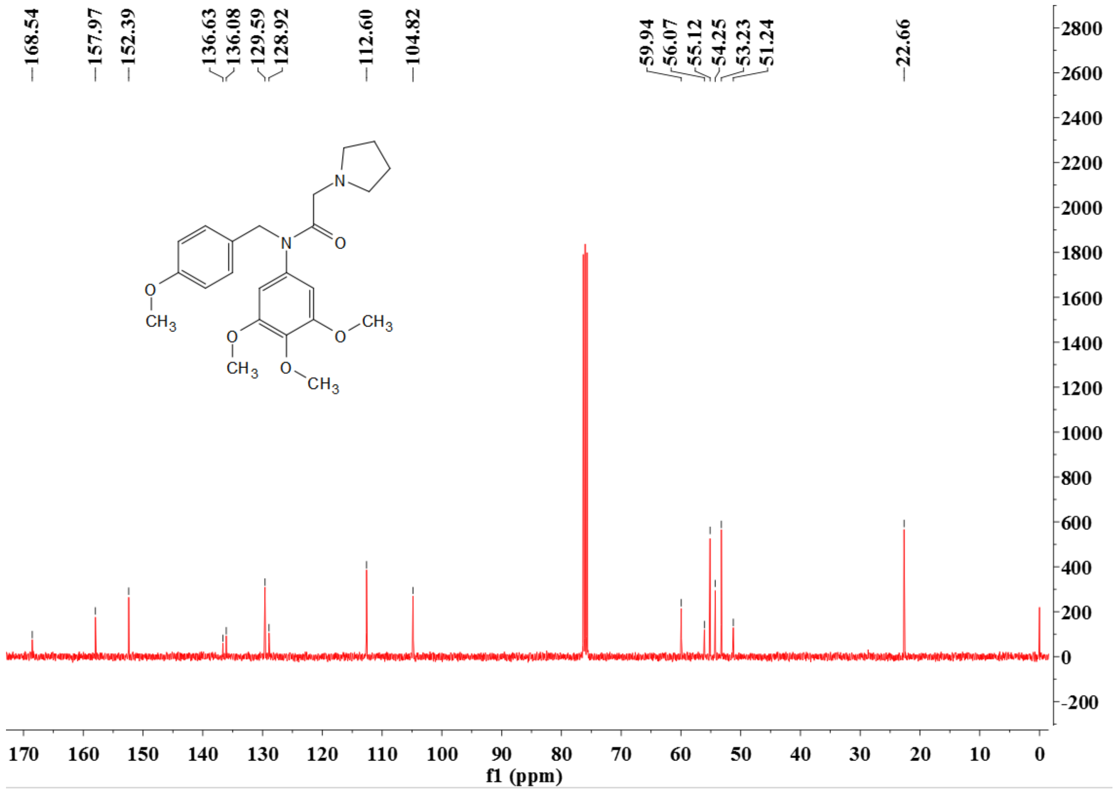

N-(4-methoxybenzyl)-2-(piperidin-1-yl)-N-(3,4,5-trimethoxyphenyl)acetamide (17e)

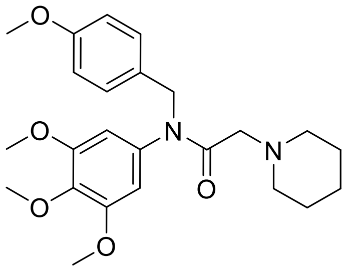

Yield: 61%. White solid, m.p.:75~77 °C. ^{1}H NMR (400 MHz, $CDCl_3$) δ 7.08 (d, *J* = 8.5 Hz, 2H), 6.72 (d, *J* = 8.5 Hz, 2H), 6.04 (s, 2H), 4.69 (s, 2H), 3.78 (s, 3H), 3.71 (s, 3H), 3.63 (s, 6H), 2.85 (s, 2H), 2.35 (s, 4H), 1.57 – 1.42 (m, 4H), 1.32 (s, 2H). ^{13}C NMR (100 MHz, $CDCl_3$) δ 168.38, 157.96, 152.34, 136.60, 136.20, 129.57, 128.93, 112.59, 104.88, 59.95, 59.30, 55.13, 54.25, 53.61, 51.28, 24.86, 22.98. HRMS (m/z) $[M + H]^+$ calcd for $C_{24}H_{33}N_2O_5$, 429.2389; found, 429.2392.

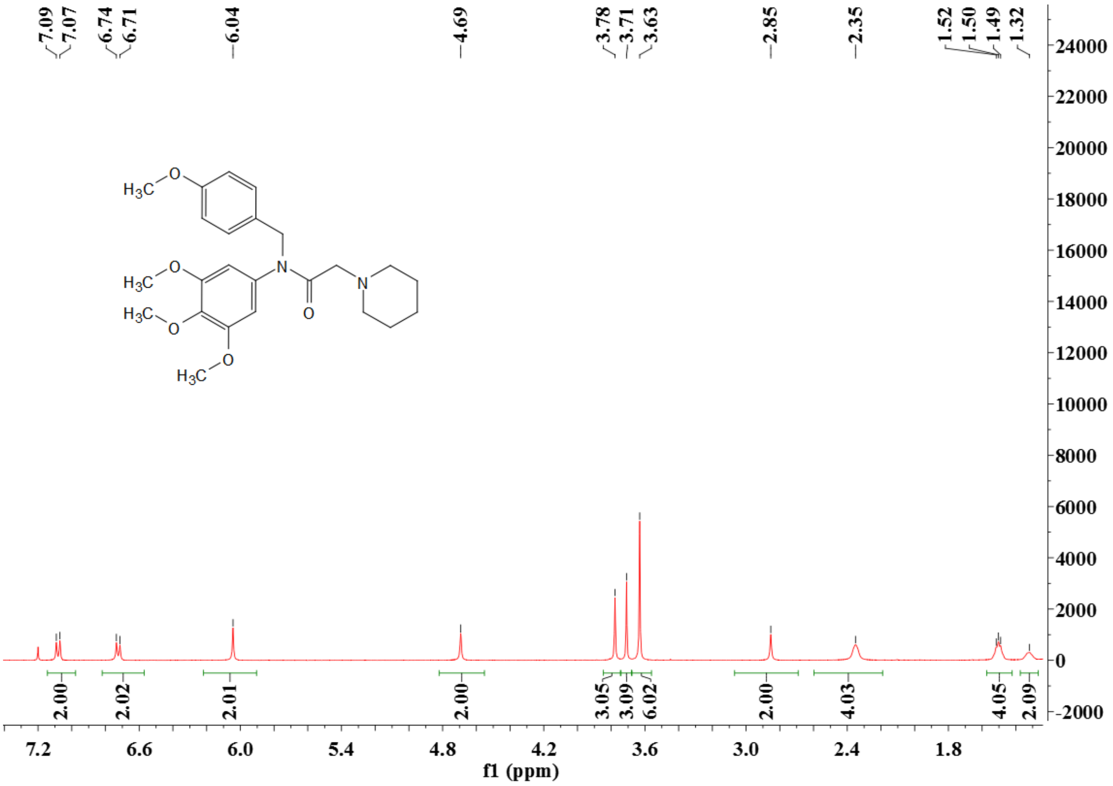

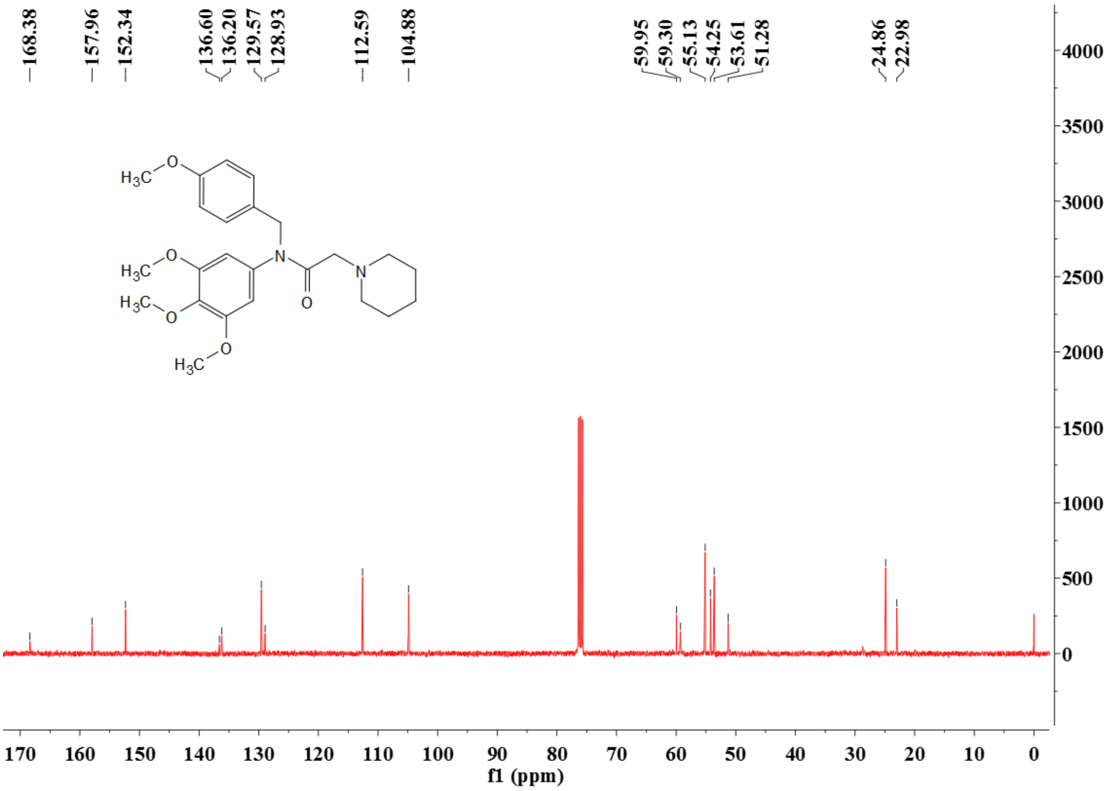

N-(4-methoxybenzyl)-2-(3-methylpiperidin-1-yl)-N-(3,4,5-trimethoxyphenyl)acetamide (17f)

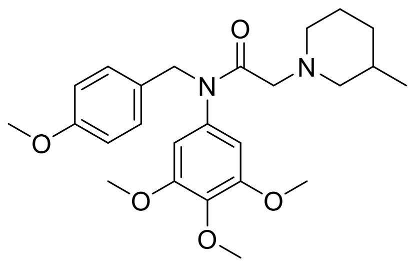

Yield: 80%. White solid, m.p.:72~74 ºC. ^{1}H NMR (400 MHz, $CDCl_3$) δ 7.08 (d, *J* = 8.5 Hz, 2H), 6.72 (d, *J* = 8.4 Hz, 2H), 6.04 (s, 2H), 4.69 (s, 2H), 3.78 (s, 3H), 3.71 (s, 3H), 3.63 (s, 6H), 2.86 (s, 2H), 2.74 (s, 2H), 1.85 (s, 1H), 1.59 (d, *J* = 10.3 Hz, 3H), 1.51 (s, 2H), 0.75 (s, 1H), 0.75 (d, *J* = 5.6 Hz, 3H). ^{13}C NMR (100 MHz, $CDCl_3$) δ 168.44, 157.97, 152.34, 136.60, 136.20, 129.58, 128.93, 112.59, 104.89, 61.00, 59.95, 59.09, 55.13, 54.25, 53.04, 51.27, 31.65, 30.06, 24.50, 18.64. HRMS (m/z) $[M + H]^+$ calcd for $C_{25}H_{35}N_2O_5$, 443.2546; found, 443.2548.

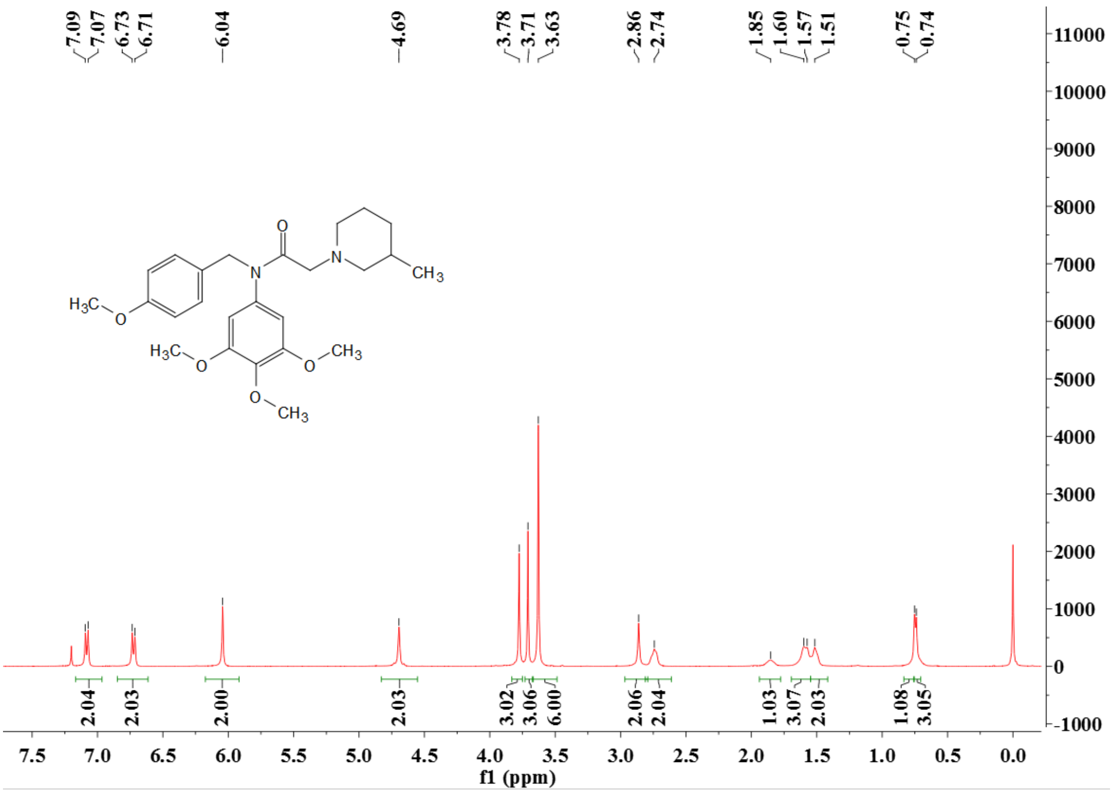

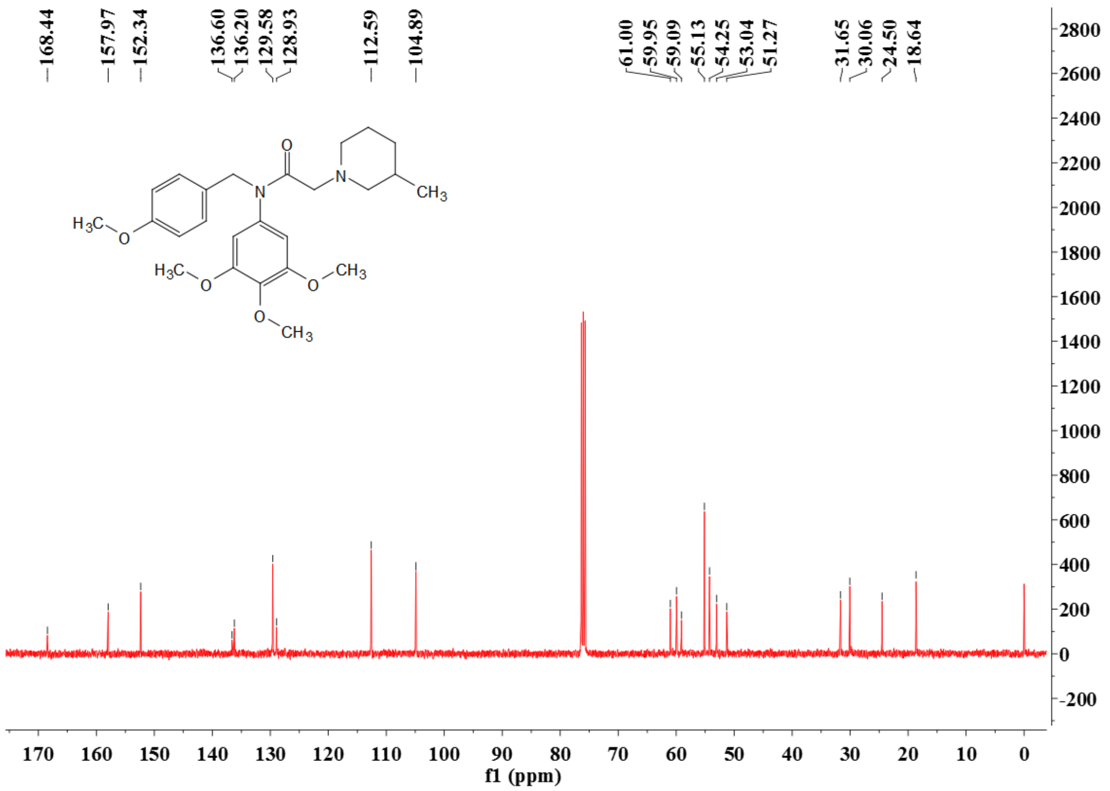

2-(3,5-dimethylpiperidin-1-yl)-N-(4-methoxybenzyl)-N-(3,4,5-trimethoxyphenyl)acetamide (17g)

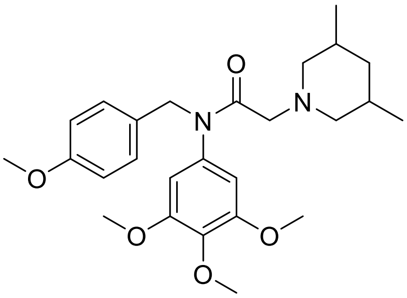

Yield: 62%. White solid, m.p.:96~98 °C. ^{1}H NMR (400 MHz, $CDCl_3$) δ 7.08 (d, J = 8.4 Hz, 2H), 6.72 (d, J = 8.4 Hz, 2H), 6.04 (s, 2H), 4.70 (s, 2H), 3.78 (s, 3H), 3.71 (s, 3H), 3.63 (s, 6H), 2.87 (s, 2H), 2.74 (d, J = 9.5 Hz, 2H), 1.61 (s, 2H), 1.58 (s, 1H), 1.48 (s, 2H), 0.74 (d, J = 6.3 Hz, 6H), 0.40 (q, J = 11.8 Hz, 1H). ^{13}C NMR (100 MHz, $CDCl_3$) δ 168.43, 157.98, 152.35, 136.61, 136.15, 129.60, 128.92, 112.59, 104.88, 60.52, 59.95, 58.79, 55.12, 54.25, 51.27, 40.79, 30.02, 18.51. HRMS (m/z) $[M + H]^+$ calcd for $C_{26}H_{37}N_2O_5$, 457.2702; found, 457.2706.

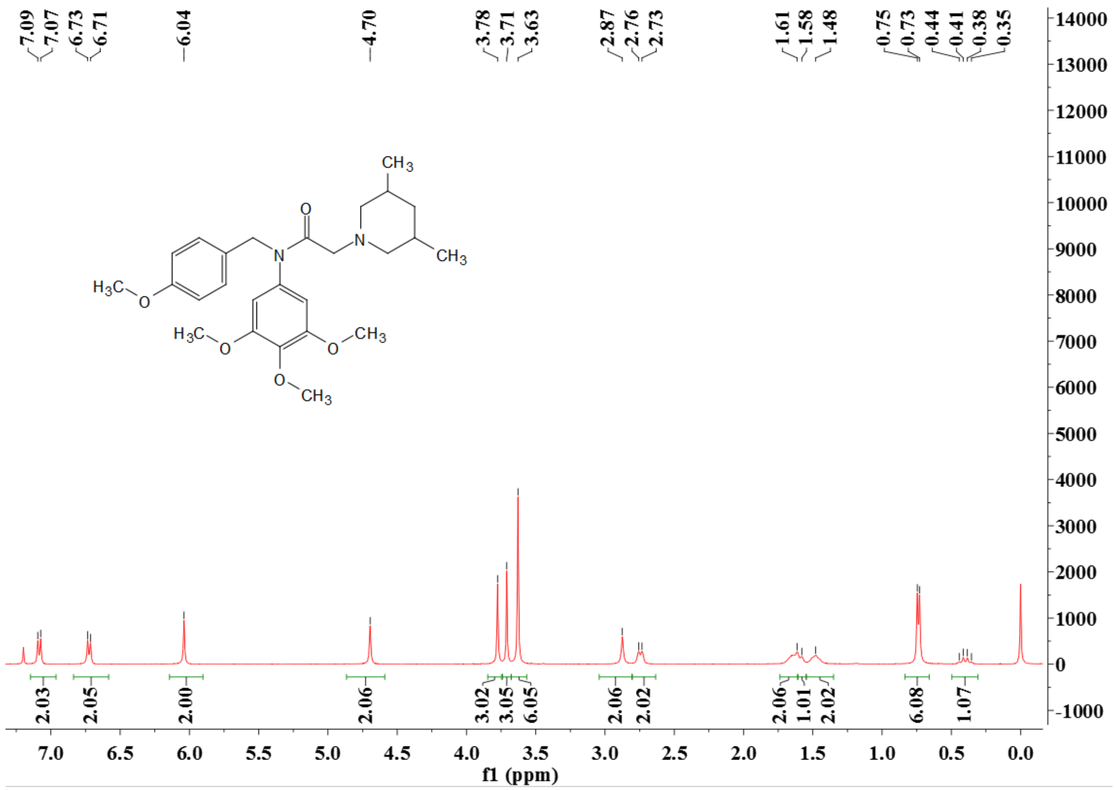
7.09
7.07
6.73
6.71
6.04
4.70
3.78
3.71
3.63
2.87
2.76
2.73
1.61
1.58
1.48
0.75
0.73
0.44
0.41
0.38
0.35
2.03
2.05
2.00
2.06
3.02
3.05
6.05
2.06
2.02
2.06
1.01
2.02
6.08
1.07
f1 (ppm)

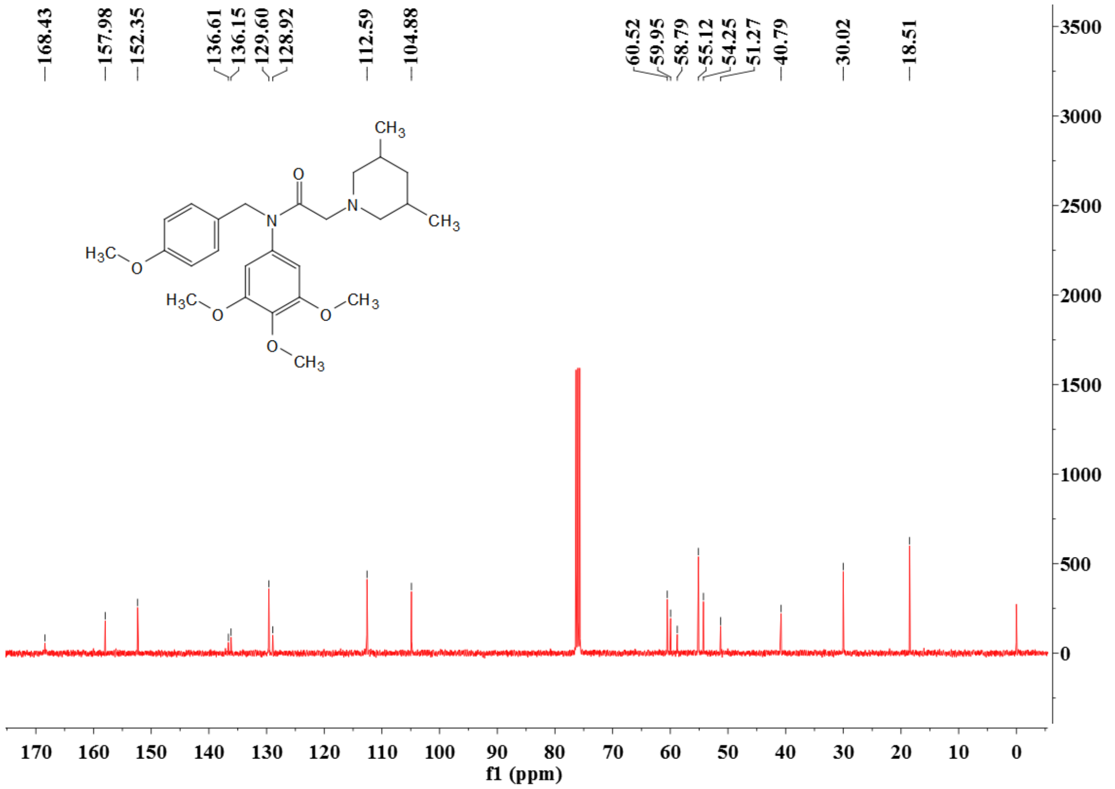
168.43
157.98
152.35
136.61
136.15
129.60
128.92
112.59
104.88
60.52
59.95
58.79
55.12
54.25
51.27
40.79
30.02
18.51
f1 (ppm)
